# A Fast Radio Map Construction Method Merging Self-Adaptive Local Linear Embedding (LLE) and Graph-Based Label Propagation in WLAN Fingerprint Localization Systems

**DOI:** 10.3390/s20030767

**Published:** 2020-01-30

**Authors:** Yepeng Ni, Jianping Chai, Yan Wang, Weidong Fang

**Affiliations:** 1 School of Data Science and Media Intelligence, Communication University of China, No.1 Dingfuzhuang East Street, Chaoyang District, Beijing 100024, China; jp_chai@cuc.edu.cn (J.C.); wy@cuc.edu.cn (Y.W.); 2 Key Laboratory of Wireless Sensor Network & Communication, Shanghai Institute of Micro-System and Information Technology, Chinese Academy of Sciences, Shanghai 201800, China; weidong.fang@mail.sim.ac.cn

**Keywords:** indoor positioning, radio map, LLE, manifold learning, graph-based label propagation

## Abstract

Indoor WLAN fingerprint localization systems have been widely applied due to the simplicity of implementation on various mobile devices, including smartphones. However, collecting received signal strength indication (RSSI) samples for the fingerprint database, named a radio map, is significantly labor-intensive and time-consuming. To solve the problem, this paper proposes a semi-supervised self-adaptive local linear embedding algorithm to build the radio map. First, this method uses the self-adaptive local linear embedding (SLLE) algorithm based on manifold learning to reduce the dimension of the high-dimensional RSSI samples and to extract a neighbor weight matrix. Secondly, a graph-based label propagation (GLP) algorithm is employed to build the radio map by semi-supervised learning from a large number of unlabeled RSSI samples to a few labeled RSSI samples. Finally, we propose a *k* self-adaptive neighbor weight (kSNW) algorithm, used for radio map construction in this paper, to realize online localization. The results of the experiments conducted in a real indoor environment show that the proposed method reduces the demand for large quantities of labeled samples and achieves good positioning accuracy. With only 25% labeled RSSI samples, our system can obtain positioning accuracy of more than 88%, within 3 m of localization errors.

## 1. Introduction

The radio map is the most important part of the WLAN fingerprint localization systems and the key to ensuring the positioning accuracy of the system. Regardless of the deterministic or probabilistic positioning algorithm, the radio map is required to provide accurate mapping from the received signal strength indication (RSSI) sample to the physical location coordinates to complete localization. Building a high-accuracy radio map requires engineers to set enough reference points (RPs) in the positioning area, collect sufficient RSSI samples at these reference points, and these RSSI samples should include the access points (AP) information in the area as much as possible. Therefore, in the indoor complex environment where APs are densely deployed, building a high-accuracy radio map is time-consuming and labor-intensive [1,2]. This high-cost radio map building method severely restricts the application and development of a WLAN fingerprint localization system.

The essence of the WLAN fingerprint localization systems is a process of pattern recognition, and the process of building a radio map is the calibrating of pattern recognition. In order to reduce the consumption of timing and labor cost when building a radio map, this paper proposes a radio map construction method merging self-adaptive local linear embedding (SLLE) algorithm [3] and graph-based label propagation (GLP) algorithm [4] based on the idea of manifold learning. It first uses the SLLE algorithm to reduce the dimension of high-dimensional RSSI and extract the neighbor weight matrix, then a GLP algorithm is employed to construct the radio map by semi-supervised learning from a large number of unlabeled RSSI samples to a few labeled RSSI samples, finally, it proposes a kSNW algorithm to realize online positioning under the radio map constructed in this paper. Our proposed method greatly improves the usability of the indoor WLAN fingerprint localization systems.

This paper is organized as follows. Section 2 briefly introduces the related work. Section 3 presents the method of radio map building by using the SLLE algorithm and GLP algorithm in detail. Section 4 describe the experimental testbed and conduction of the experiment, and then the experimental results are analyzed and compared. Conclusions are drawn in the last section.

## 2. Related Work

With the increasing importance of location-based services, the construction of radio maps in indoor environments has gradually formed a research point, especially in terms of reducing time-consumption and labor costs. This includes methods based on crowdsourcing [5,6], semi-supervised learning [7,8,9] or unsupervised learning [10,11], the path loss model [12,13], interpolation [14,15], and the merging algorithm [16]. These methods generally reduce the cost of building a radio map. We will discuss some representative works here.

The fundamental idea of crowdsourcing refers to allocating a workload to several participants, in this case, including both professional surveyors and general users. Molé [5] and FreeLoc [6] have been proposed to promote users to measure fingerprints with locations or semantic labels (e.g., corridors, hallways, and rooms). Nevertheless, users are commonly reluctant to give precise location labels for privacy considerations, significantly lowering the built radio map performance.

The authors in [7,8,9] employed semi-supervised methods, which is also the core method of this paper. They [7,9] reduced the high-dimensional RSSI to two-dimensions through manifold alignment to obtain the position information. However, this method will reduce the positioning accuracy, as the dimension is fixed from the beginning. The researchers in 8 developed a semi-supervised learning algorithm, termed Co-Forest, creating and repeatedly refining a random forest ensemble classifier that exhibits high performance to estimate locations. However, it requires considerable location-labeled fingerprints to start the learning, so a long period is taken.

For reducing the calibration work, the authors in [10,11] employed a radio propagation model and Hidden Markov Model (HMM) for rapidly implementing an indoor positioning mechanism. Giving several independence assumptions, it adopts a distribution of discrete probability for expressing all hypothetical positions, and such a probability distributing process is only advanced when novel RSSI is collected or a user is moved. The position is estimated by weighting the different hypotheses. Such an approach requires high computation overhead on the user terminal, and the accuracy is relatively low, as the radio propagation model is not capable of modelling the realistic environment appropriately.

In [12], the system employed Weibull distribution to build the path loss model for the distribution of the RSSI over time. By inheriting the updating method from [11], the authors in [13] presented a novel algorithm to reconstruct a radio map by clustering the path-loss parameters of each reference point. However, both of them hardly describe the RSSI fluctuating sample due to the complex indoor propagation environment.

The researchers in [14,15] used the inverse distance weighted (IDW) and Kriging methods, respectively, which are most widely used for building radio maps with approximate positioning accuracy. The authors in [14] showed that IDW interpolation and extrapolation methods can improve both the horizontal positioning accuracy and the floor detection probability. The researchers in [15] presented an appropriate spatial interpolation method, which studied the signal propagation characteristic and applied it to an interpolated database with the Kriging algorithm. These interpolation methods can achieve good positioning accuracy with a small enough sampling interval and a uniform sampling density. When the sampling interval is large and there are many APs, their performance will become poor.

The authors in [16] proposed an approach of radio map construction by incorporating crowdsourcing, interpolation, and the path loss model. Such an approach is capable of acquiring the identical positioning accuracy under sparse RP intervals set as 9.6 m, as the complete manual radio map with the interval at 1.2 m. However, such an approach ignores the walls attenuation and device heterogeneity, that makes it difficult to use in a real environment.

## 3. Proposed Method Merging SLLE and GLP

### 3.1. Feasibility Analysis of RSSI Sample Semi-Supervised Manifold Learning

There is an assumption in manifold learning that the processed data is sampled on a potential manifold or that there is a potential manifold for this set of data. Different methods have different requirements for the properties of the manifold, which also leads to the assumption of different properties of the manifold. The local linear embedding (LLE) algorithm assumes that the sampled data resides is locally linear in the low-dimensional manifold, and each sampling point can be linearly represented by its nearest neighbors. Similar to manifold learning, in graph-based semi-supervised learning methods, there are certain assumptions about the internal relationships of processed data. For example, the GLP algorithm hopes that the data meets that: points with similar characteristics tend to have the same label. Whether the semi-supervised learning or the manifold learning, all of them have potential assumptions on the sample data. To achieve a good learning effect, the sample data must meet the assumptions.

In order to verify whether the RSSI in the indoor complex environment meets the assumptions of LLE and graph-based semi-supervised learning, we built a radio map of the office environment, and select five adjacent reference points from the radio map. As shown in Figure 4 (in Section 4.1), the five adjacent reference points are denoted as $l_{m}$, $l_{e}$, $l_{s}$, $l_{w}$, and $l_{n}$. $l_{m}$ is the middle reference point, and $l_{e}$, $l_{s}$, $l_{w}$, and $l_{n}$ are the adjacent reference points of east, south, west, and north, respectively. The reference point interval is 2 m. A total of 13 APs are deployed in the office. Table 1 shows the RSSI obtained by sampling these reference points.

First, we verify whether the RSSI samples meet the assumption of LLE. Taking $l_{m}$ as an example, it can be clearly seen from Table 1 that the RSSI of each AP sampled at $l_{m}$ can be linearly represented by the RSSI sampled at the remaining four RPs, which satisfies the assumptions of the LLE algorithm. We then verify whether the RSSI samples meet the hypothesis conditions of graph-based semi-supervised learning. We also observe the RSSIs of the five reference points in Table 1, the RPs that are adjacent to each other, have approximate RSSIs. Conversely, the physical locations corresponding to two similar RSSIs should also be similar. The RSSI distribution characteristics are in line with the graph-based semi-supervised learning hypothesis.

### 3.2. Method Design

The RSSI collected in the indoor complex environment contains information of multiple APs, which can easily form a Curse of Dimensionality [17], which increases the complexity of subsequent semi-supervised learning and positioning. According to the conclusions in the previous section, LLE can be used to find low-dimensional manifolds embedded in the high-dimensional RSSI sample space to achieve dimensionality reduction. The LLE algorithm is a dimensionality reduction method that recovers the non-linear structure of high-dimensional data from local linear fittings. LLE maps high-dimensional inputs to a unified low-dimensional coordinate system. The optimization does not involve local minimization. When the sample data meets the LLE assumption, the algorithm can obtain the global optimal solution.

Given a set of RSSI samples $X=\left\{x_{1},\;x_{2},\cdots ,x_{n}\right\}$, $x_{i}\in R^{D}$, is composed of N samples which have D dimension vectors. Every sample is sampled from a potential manifold. By calculating the Euclidean distance between all sample points, we can determine the k nearest neighbors for each sample point.

The selection of the parameter k plays a key role in the LLE algorithm. If k is too large, the LLE cannot reflect local characteristics, may affect the smoothness of the entire manifold, and may even lose some small-scale structures of the manifold. If k is too small, the LLE cannot maintain the topological structure of the sample points in low-dimensional space. The LLE algorithm hopes that the data density is approximately same in the observation space, to reduce the impact of the parameter k; however, it is difficult to ensure that the sampling density of high-dimensional data is consistent in practical operations. Therefore, it is not reasonable to use a fixed value of k for the nearest neighbor selection.

To overcome the problem, we propose a self-adaptive k method based on the genetic algorithm [18] procedure to optimize the performance of the LLE algorithm. The steps are as follows:

(1) Chromosome coding

Take the parameter *k* as the chromosome, if there are *N* high-dimensional data, we can assume that the value interval of k is [1, $\sqrt{N-1}$] according to experience.

(2) Initialization

The population number *M* is $\left(\sqrt{N-1}\right)$, and other genetic algorithm parameters do not need to be set, because the search for k value will start from 1 at this time until the termination condition is met.

(3) Fitness Function

The k is proportional to the data density. In areas with a high data density, i.e., the more neighboring points required to reflect the local geometric relationship of the sample points, the k is larger. The fewer the required neighboring points, the k is smaller. Data density change can be measured by the squared Euclidean distance between the sample point $x_{i}$ and k nearest neighbor point $x_{j}$. Let $\beta_{i\_max}$ be the maximum Euclidean distance between the sample point and k nearest neighbors:

(1) $$\beta_{i{\_}_{max}}\left(k\right)=\mathrm{MAX}||x_{i}-x_{j}||{}^{2}.$$

Let $\beta_{i\_sum}$ be the sum of the Euclidean distances between the sample points and k nearest neighbors:

(2) $$\beta_{i\_sum}\left(k\right)=\sum_{j=1}^{k}||x_{i}-x_{j}||{}^{2}.$$

Then let $\rho_{i}$ be the data density change of the sample point $x_{i}$:

(3) $$\rho_{i}\left(k\right)=\frac{\beta_{i\_max}}{\beta_{i\_sum}}.$$

$\rho_{i}$ represents the proportion of the Euclidean distance between $\beta_{i\_max}$ and $\beta_{i\_sum}$. When k=1, it means that only one neighbor point is taken, then $\rho_{i}=1$. In the case where the data density does not change drastically, as k continues to increase, $\rho_{i}$ continues to decrease, but once the data density decreases, that is, $\beta_{i\_max}$ greatly increases, $\rho_{i}$ will appear as an inflection point. We chose the value of k at the inflection point as the most suitable parameter for the sample point $x_{i}$, and it is recorded as $k_{i}$.

**Figure 1:**
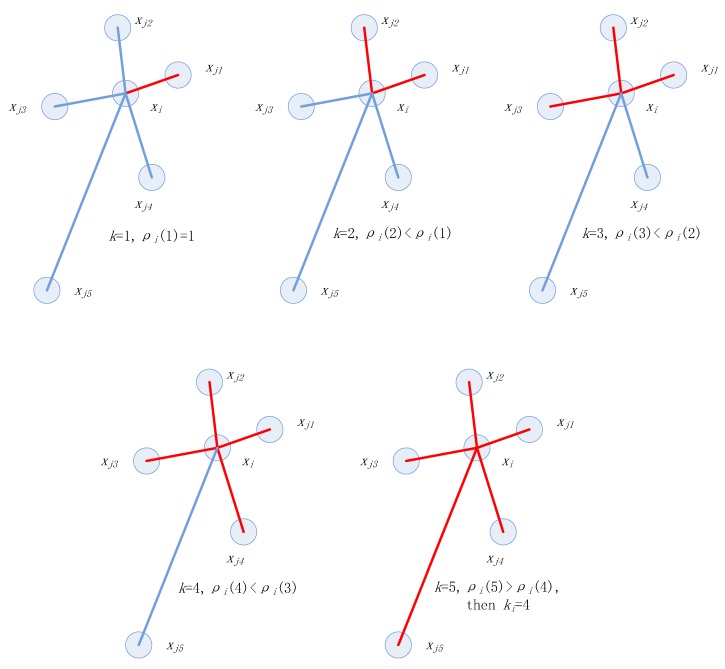
The variation of data density with different choices of parameter k.

Figure 1 shows the variation of data density. Suppose $x_{i}$ has five nearest neighbors denoted as $x_{j1}$, $x_{j2},x_{j3},x_{j4},\;\mathrm{and}\;x_{j5}$, and their Euclidean distance to $x_{i}$ increases ascending. The five sub-graphs show the change of $\rho_{i}\left(k\right)$ when the parameter *k* is increasing. The inflection point of the data density appears when k=4.

Therefore, we can use Equation (3) as the fitness function and select the best fit k with the termination condition.

(4) Individual evaluation

$\rho_{i}\left(k\right)$ of the sample point $x_{i}$ will be recorded as an individual evaluation.

(5) Termination condition

**Figure 2:**
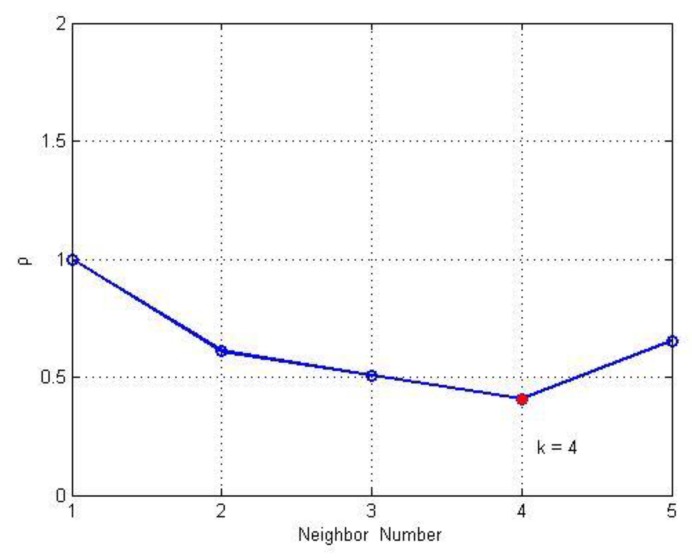
Data density inflection point diagram.

The search is terminated when an inflection point occurs in $\rho_{i}\left(k\right)$. That is, when $\rho_{i}(k-1)>\rho_{i}(k)$ and $\rho_{i}(k)<\rho_{i}(k+1)$, $k_{i}=k$. The search result is shown in Figure 2.

After searching for the parameter k of each RSSI sample in sequence, we obtained the best fit $k_{i}$ for $x_{i}$. Then we can select the nearest neighbor of $x_{i}$ for local linear embedding according to $k_{i}$.

LLE hopes that each sample point and its neighboring points have local linear structural features. Using linear coefficients to describe this local geometric feature, each sample point can be reconstructed through its neighboring points. This reconstruction error can be calculated by the following cost function [19]:

(4) $$\varepsilon \left(W\right)=\;\sum_{i=1}^{N}x_{i}-\sum_{j=1}^{k_{i}}w_{ij}x_{j}{}^{2}.\;$$

ε(W) is the sum of the squared distances of sample points and their reconstruction, where $w_{ij}$ is the weight of the nearest neighbor point $x_{j}$ of the sample point $x_{i}$ when reconstructing $x_{i}$. To minimize the cost function, we propose two constraints: First, each sample point is reconstructed only by its $k_{i}$ nearest neighbors. If $x_{j}$ does not belong to the $k_{i}$ nearest neighbors of $x_{i}$, let $w_{ij}$ = 0. Second, the sum of each row of the weight matrix should be equal to 1, that is, $\underset{j}{\sum }w_{ij}=1$. As $k_{i}$ is different for each sample point, the weight matrix must be created according to the maximum value of $k_{i}$, and the blank parts are filled with zero.

Consider any RSSI sample point x, whose k neighbor points are $\eta_{j}$ and the sum of its reconstruction weights $W_{j}$ is 1. We can write the reconstruction error as:

(5) $$\varepsilon =||x-\sum_{j=1}^{k}W_{j}\eta_{j}||{}^{2}=||\sum_{j=1}^{k}W_{j}{\left(x-\eta_{j}\right)||}^{2}=\sum_{\mathrm{jk}}W_{j}G_{jk}W_{k}$$

where $G_{jk}$ is the covariance matrix:

(6) $$G_{jk}=\left(x-\eta_{j}\right)\left(x-\eta_{k}\right).$$

$G_{jk}$ has characteristics of symmetry and is positive semi-definite due to its construction method. Therefore, we can analyze the minimization problem of reconstruction error by the Lagrange multiplier under the constraint of $\underset{j}{\sum }w_{ij}=1$. According to the inverse of the covariance matrix, the optimal weight can be given by:

(7) $$W_{j}=\frac{\sum_{k}G_{jk}^{-1}}{\sum_{\mathrm{lm}}G_{lm}^{-1}}.$$

If Equation (7) has a unique solution, then the covariance matrix G should be a non-singular matrix. If G is a singular matrix in actual operation, then G must be regularized. The specific method is to add a small multiplier to the matrix. At this point we can calculate the reconstruction weights.

In order to minimize the reconstruction error, the weight matrix W must obey an important symmetry: For all specific sample points, after undergoing various transformations such as rotation, rearrangement, and transformation between them and their nearest neighbors, the topological structure between them must remain unchanged so that the reconstruction weights can accurately describe the basic geometric characteristics of each neighbor. Therefore, it can be considered that the local geometric features of the data in the high-dimensional original space and the local topology on the low-dimensional manifold after the mapping are completely equivalent. Then, we can use the obtained weight matrix to reconstruct in a low-dimensional space and work out the low-dimensional embedding Y, by minimizing the reconstruction error.

Before mapping X to Y, we need to determine the dimension d of the low-dimensional space. At present there have been some studies on intrinsic dimension estimation of high-dimensional data [20,21,22]. Considering the complexity of the LLE algorithm and its method of using Euclidean distance to determine the nearest neighbors, we use a method similar to principal component analysis (PCA) to find the intrinsic dimension [23]. When calculating the reconstruction weight matrix of each sample point, the LLE algorithm must construct a local covariance matrix $G_{i}$, so the output dimension of the sample point can be calculated by the following formula.

(8) $$\frac{\sum_{j=1}^{d}\lambda_{j}}{\sum_{j=1}^{k}\lambda_{j}}\geq \theta^{*}.$$

$\lambda_{j}$ is the eigenvalue of $G_{i}$, and is arranged in descending order. $\theta^{*}$ is the threshold value of the projection space retention information, and usually takes a value greater than 80%. Equations (5)–(8) consider that the ratio of the sum of the top d eigenvalues to the sum of all eigenvalues is not less than 80%, which can satisfy the low-dimensional embedding of the original data information. Each sample point needs to calculate the output dimension, and the average value of the output dimensions of all sample points is specified as the output dimension of the sample space.

After determining the weight matrix W and the output dimension d, we rewrite the cost function of the reconstruction error as follows:

(9) $$\phi \left(Y\right)=\;\sum_{i=1}^{N}||y_{i}-\sum_{j=1}^{k_{i}}w_{ij}y_{j}||{}^{2}.\;$$

Equation (9) is similar to Equation (4), but the weight $w_{ij}$ is fixed at this time, and the low-dimensional coordinate $y_{i}$ needs to be optimized. In order to limit the uniform distribution of low-dimensional data and prevent the data set from collapsing to the coordinate origin in low dimensions, we added two constraints to Y: $\sum_{i=1}^{N}y_{i}=0$ and $\frac{1}{N}.\sum_{i=1}^{N}y_{i}y_{i}{}^{T}=I$, where I is a N-dimensional identity matrix. Under such constraints, the problem of minimizing reconstruction errors in low-dimensional space can be simplified as:

(10) $$\min\phi \left(Y\right)=\sum_{i=1}^{N}||YI_{i}-YW_{i}||{}^{2}=\sum_{i=1}^{N}||Y{\left(I_{i}-W_{i}\right)||}^{2}=\mathrm{tr}\left(YMY^{T}\right).$$

$I_{i}$ represents the ith column of the identity matrix I, and $M={\left(I-W\right)}^{T}\left(I-W\right)$. Using the Lagrange multiplier method again, combined with constraints, the solution is $MY^{T}=\lambda Y^{T}$. To minimize the value of the cost function, take the eigenvector corresponding to the minimum d non-zero eigenvalues of M as the low-dimensional embedded coordinate Y. During the processing, the eigenvalues of M are arranged ascending, and the first eigenvalue is almost close to zero, so the first eigenvalue is discarded. Usually, the eigenvectors corresponding to the eigenvalues between 2∼(d+1) are taken as the output results.

**Figure 3:**
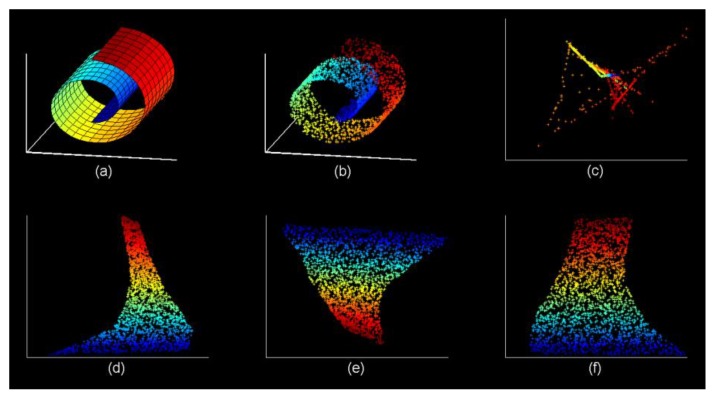
The effect with different choices for parameter *k* on dimensionality reduction performance: (**a**) Swiss roll, (**b**) 2000 sampling points, (**c**) *k* = 4, (**d**) *k* = 12, (**e**) *k* = 36, (**f**) self-adaptive *k.*

In order to compare the performance of the traditional LLE algorithm with our proposed parameter *k* adaptive LLE algorithm, we use them to reduce the dimensionality of the Swiss volume graph, and the results are shown in Figure 3. It can be clearly seen that the choice of parameter k has a great impact on the dimensionality reduction results. As we discussed before, if the k is inappropriate, the expansion of high-dimensional data in low-dimensional space will shrink or deform, and the local linear structure of high-dimensional data cannot be retained. Due to the diversity of data, the k is difficult to grasp, and most of the time it is set based on human experience. In contrast, for our proposed self-adaptive LLE algorithm, as it can adaptively select the k according to the data density of each sample point, its dimension reduction effect is significantly better than the traditional LLE algorithm. As shown in Figure 3f, we fully expand the 3D graphics in the 2D space.

After obtaining a low-dimensional RSSI set, we will use the GLP algorithm to perform semi-supervised learning on this data set. The data set will be redefined below. The letters and labels used in the definition have nothing to do with the previous content.

The RSSI set contains N samples, of which the first l is labeled data and the rest is unlabeled data. The labeled data can be recorded as $\left\{\left(x_{1},y_{1}\right)\cdots \left(x_{l},y_{l}\right)\right\}$, where $y\in \left\{C_{1},C_{2},\cdots ,C_{m}\right\}$ is the label set of the data, we assume that all labels are already known and all appear in the labeled data. Unlabeled data can be written as $\left\{x_{l+1},\cdots ,x_{l+u}\right\}$, where l+u=N. Next, we will respectively use L and U to represent labeled and unlabeled data. Our mission is to use the GLP algorithm to predict the labels of the data in ***U*** through the label information in L.

The GLP algorithm is a graph-based semi-supervised learning algorithm. We need to use the relationship of sample data to build a fully connected graph. The nodes of the graph are data points and contain all labeled and unlabeled data. The edges of nodes i and j in the graph represent the similarity of the two nodes. The weight of the edges between nodes is proportional to the similarity of the nodes. The edge weights of nodes i and j are defined as follows:

(11) $$w_{ij}=\exp\left(-\frac{||x_{i}-x_{j}||{}^{2}}{\alpha^{2}}\right)$$

where α is the hyper-parameter and is used to control the weight $w_{ij}$. After getting the weights of all edges, we can propagate labels through the edges between nodes. The greater the weight of the edge, the easier for the label to propagate. For label propagation, we define an N×N probability transition matrix P:

(12) $$P_{ij}=P\left(i\to j\right)=\frac{w_{ij}}{\sum_{k=1}^{N}w_{ik}}$$

$P_{ij}$ represents the transition probability from node i to node j. Then we define a label matrix $Y_{L}$ with l rows and M columns, and the i-th row represents the label indication vector (i∈l) of the labeled sample data $\left(x_{i},y_{i}\right)$, that is, if the i-th sample is classified as j, The j-th element of the row is 1, the others are 0. Similarly, we also define a u×M matrix $Y_{U}$ for u unlabeled sample data. After merging the two matrices, we have an N×M matrix $F=\left[Y_{L};Y_{U}\right]$. Each row in the matrix F represents the classification probability distribution of a sample data. For unlabeled data, we randomly initialize the row it represents, as long as the sum of each row is 1. The GLP algorithm steps are as follows:

(1) Execution propagation: F←PF.
(2) Lock the marked data by replacing the first l rows of F with $Y_{L}$: $F_{L}=Y_{L}$.
(3) Repeat steps (1) and (2) until F converges.
(4) Assign labels to unlabeled data according to F.

Step (1) is to left-multiply matrix P by matrix F, so that each node propagates its own label to other nodes with probability P. The similarity between the two nodes is directly proportional to the probability of their label propagation. Step (2) is very important, as we need to keep the original label data unchanged, so each time the execution of the propagation is completed, we need to restore $F_{L}$ to the original label. As the label data continues to propagate its labels, the class boundary will pass through the high-density area and stay in the low-density interval in step (3). In the last step we assign labels based on the specific application.

In the process of the GLP algorithm, we found that after calculating F in step (1), step (2) is needed to lock the labeled data $Y_{L}$. In fact, what we really care about is the change of the label in the $Y_{U}$ part, so we can simplify the steps by calculating only the $Y_{U}$ part. First, we re-divide the matrix P:

(13) $$P=\left[\begin{array}{c} P_{LL}\\ P_{UL} \end{array}\;\begin{array}{c} P_{LU}\\ P_{UU} \end{array}\right].$$

Then there are:

(14) $$F_{U}\leftarrow P_{UL}Y_{L}+P_{UU}F_{U}.$$

We iterate Equation (14) until convergence. It can be seen from Equation (14) that the label distribution $F_{U}$ of unlabeled data depends not only on the label of the labeled data and its transition probability, but also on the current label of the unlabeled data and its transition probability, thus, this is a kind of semi-supervised algorithm using unlabeled data learning.

### 3.3. Radio Map Construction by Proposed Method and Online Positioning

#### 3.3.1. Radio Map Construction

In the indoor WLAN area, we obtained a small number of location fingerprints (labeled data) and a large number of high-dimensional RSSI samples. The location fingerprints consist of RSSI samples and their corresponding physical location. The individual RSSI samples are unlabeled data. Our task is to use the method proposed in this paper to reduce the dimensionality of RSSI samples, and predict the physical location corresponding to each unlabeled RSSI sample through the physical location information of a small number of location fingerprints.

When collecting individual RSSI samples and location fingerprints, we need to pay attention to two things. The first is to ensure that the data dimensions of all RSSI samples are equal, which is a necessary condition for using the LLE algorithm for dimensionality reduction. Due to the interference in a complex indoor environment, it is difficult for us to observe the RSSI of all APs at each location. In this case, we can record the RSSI of the missing AP as −99 dBm, so that all RSSI samples have the same dimension. The second is to try to ensure that the radio map can cover the entire localization area. In the GLP algorithm we assume that all labels are known and appear in the labeled data. In a radio map, the physical location is the data label, but unlike the traditional classification problem, we cannot collect the location fingerprints for all physical locations. In order to satisfy the assumption of the GLP algorithm, we need to collect the position fingerprint in a sparse but full-coverage manner, which can be achieved by choosing a larger RP interval when building a radio map.

We assume that there are N samples in the RSSI sample set, where the physical locations of the first m are known, and the physical locations of the remaining samples are unknown. The i-th sample can be expressed as $\left(x_{i},l_{i}\right)$, where $x_{i}\in R^{D}$ represents a D-dimensional vector, and $l_{i}\in \left\{C_{1},C_{2},\cdots ,C_{j}\right\}$ is a known physical location label. The steps to construct a radio map using the method proposed in this paper are as follows:

(1) For each RSSI sample $x_{i}$, use the self-adaptive method to calculate its most suitable neighbor number $k_{i}$.
(2) The $k_{i}$ nearest neighbor samples are obtained by comparing the Euclidean distance between $x_{i}$ and other samples.
(3) The SLLE algorithm is used to reduce the dimension of the RSSI sample $x_{i}$, to obtain its low-dimensional embedding $y_{i}$.
(4) Replace the high-dimensional data with low-dimensional data to establish a new sample set.
(5) Use the GLP algorithm to label the physical location of $y_{i}(i>M)$ and get the N×M matrix F. Each row in the matrix F represents the probability of a low-dimensional sample $y_{i}$ appearing at a physical location. The probability distribution of $y_{i}$ is $\left\{p_{i1},p_{i2},\cdots ,p_{ij}\right\}$, and this satisfies $\sum_{j=1}^{M}p_{ij}=1$.
(6) The weighted sum of the probability distribution of $y_{i}$ is used to estimate its physical location:
  (15) $$l_{i}=\left(C_{1}\times p_{i1}\right)+\left(C_{2}\times p_{i2}\right)+\cdots +\left(C_{j}\times p_{ij}\right)=\sum_{j=1}^{M}C_{j}.p_{ij}.$$

#### 3.3.2. Online Positioning

During online positioning, the system collected a high-dimensional RSSI, and needed to reduce the dimensionality before using the propagation algorithm for localization. However, due to the limitation of the LLE algorithm principle, we must reduce the dimensionality of the newly collected RSSI and the original RSSI together to maintain the integrity of the manifold. It is not economical to perform dimension reduction and label propagation learning for each localization. Aiming at this problem, and considering the linear relationship between high and low-dimensional data and its physical location, this paper proposes an algorithm that uses the corresponding position labels of low-dimensional data and neighbor weights to achieve localization, bypassing the problem of dimensionality reduction of new RSSI.

(1) Use the self-adaptive method to calculate the most suitable neighbor number k for the RSSI samples collected online.
(2) Find the k nearest neighbor sample points by comparing the Euclidean distance between $x_{i}$ and other sample points.
(3) Construct the weight $W_{j}$ for $x_{i}$ and its neighbors according to the SLLE algorithm.
(4) Use $W_{j}$ and location labels corresponding to known low-dimensional data to estimate the location to be measured $l_{x}$, $l_{x}=\sum_{j=1}^{k}W_{j}.l_{j}$.

As the location is obtained by multiplying the positions of the self-adaptive k neighbors by the neighbor weights, it is called k self-adaptive neighbor weights algorithm (kSNW).

## 4. Experiments and Discussion

### 4.1. Experimental Testbed Introduction

**Figure 4:**
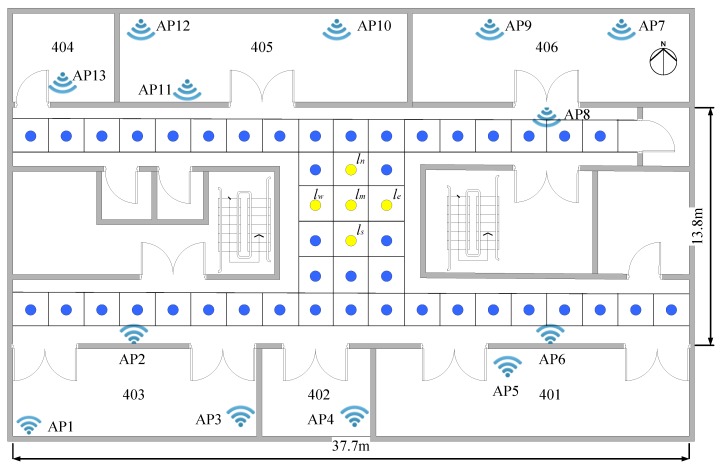
The layout of the fourth floor of the YiFu building.

**Figure 5:**
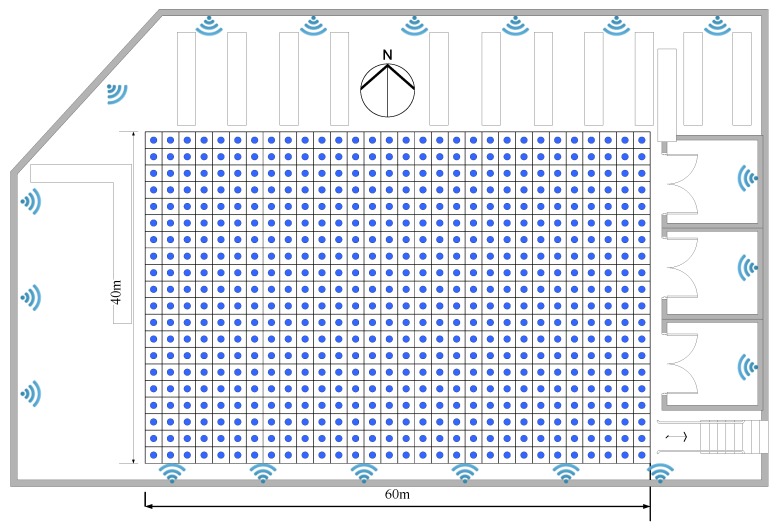
The layout of the library area.

The fourth floor of YiFu building and the DaYueCheng library were selected as the experimental scene. We deployed 13 APs on the 4th floor of the YiFu building, and a radio map was built with 48 RPs and a 2 m interval. The RSSI fingerprint of each RP is a 13-dimensional vector, as shown in Figure 4. We deployed 19 APs in the library area, and the RP interval was also 2 m. There were 600 RPs in the radio map. The RSSI fingerprint of each reference point is a 19-dimensional vector, as shown in Figure 5. The RSSI collected equipment for the above two experimental testbeds is XIAOMI HM2. In order to test the method proposed in this paper, we will use these two radio maps for dimensionality reduction and positioning experiments. The performance of the algorithm is evaluated by comparing the effect of the data dimension on the complexity of the algorithm, the impact of the amount of labeled data on the positioning accuracy, and the positioning accuracy is obtained by different positioning algorithms.

### 4.2. Algorithm Performance Test and Analysis

#### 4.2.1. Dimensionality Reduction Performance

We use the SLLE algorithm proposed in this paper to reduce the dimensions of two radio maps and analyze the complexity of the k nearest neighbors algorithm (KNN) [24] when the RSSI has different dimensions. The experimental results are shown in Table 2. The fourth column in the table refers to the complexity when using the KNN algorithm for positioning, and the complexity can be expressed as O(dN), where d represents the sample dimension and N represents the sample size, indicating that the complexity is mainly related to the sample dimension and sample size.

As can be seen from Table 2, both radio maps have achieved better dimensionality reduction results using the SLLE algorithm. At the same time, we found that although the RSSI dimension of the library area before the dimensionality reduction was higher than fourth floor of the YiFu building, the eigen dimension after the dimensionality reduction became smaller. This happens because, compared with the library area, the signal propagation in a narrow indoor environment is more complicated, and a larger eigen dimension is required to more accurately express its signal characteristics.

#### 4.2.2. Localization Performance

This section uses the reduced-dimensional RSSI samples to test the performance of the label propagation algorithm. Considering the sample size, the experiment only selects the radio map of the library area. The experiment is designed as follows: The established radio map of the library area contains the location fingerprints of 600 RPs, we took the fingerprints of 50, 100, 150, and 300 RPs, according to Table 3. Correspondingly, 550, 500, 450, and 300 RSSI samples were randomly collected by volunteers as unlabeled samples to form five data sets of the same sample size. Through semi-supervised training of DS2~5 with GLP algorithm, we built five radio maps (RM1–5) with the same density.

**Figure 6:**
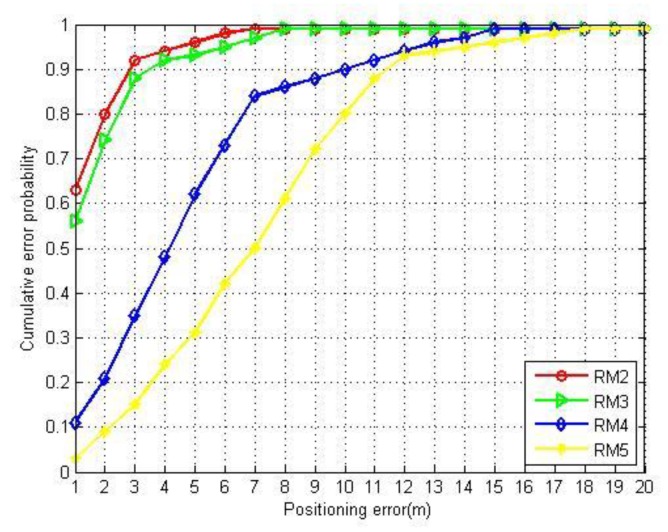
The positioning errors under different proportions of labeled fingerprints.

Next, we used the kSNW algorithm and RM2–5 to conduct a positioning test. Figure 6 shows the positioning accuracy under different proportions of labeled fingerprints. When the number of labeled fingerprints increases, the positioning accuracy also improves. When the proportion of labeled fingerprints reaches 25%, the probability of errors within 3 m using the kSNW algorithm is close to 90%. When the proportion of labeled data continues to increase, although the positioning accuracy is still improving, the range of change is small. In order to balance positioning accuracy and labor cost, we determine that the proportion between labeled sample size and total sample size is satisfied when it is up to 25%.

**Figure 7:**
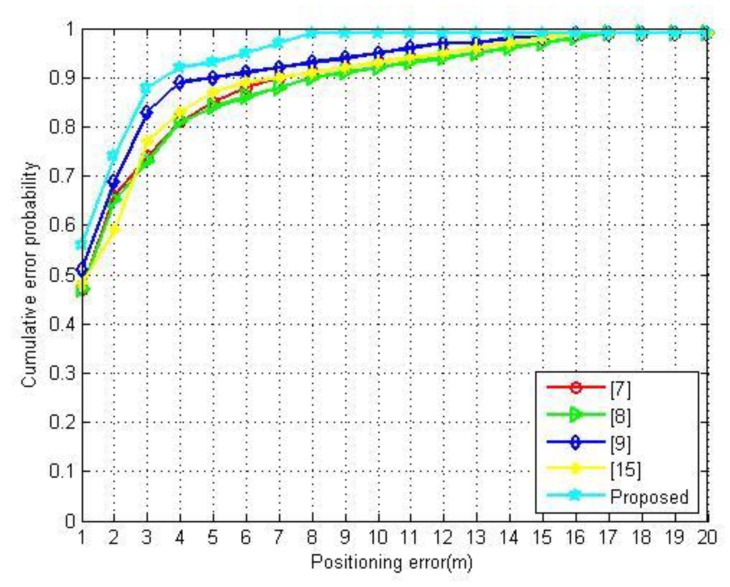
The positioning errors using different methods.

Next, the merits of the established approach are shown in the positioning performance following the comparison of estimated locations with those in [7,8,9,15]. In line with the DS3 dataset, the datasets of [7,8,9,15] are adopted for constructing the radio map, separately. Figure 7 illustrates the positioning errors by different radio maps. The proposed approach outperforms the others for positioning accuracy. The probabilities of errors within 3 m by the proposed radio map is 88.30%, which is 5.30%, 11.20%, 14.19%, and 15.30% higher than the one by [9], [15], [7], and [8], respectively.

**Figure 8:**
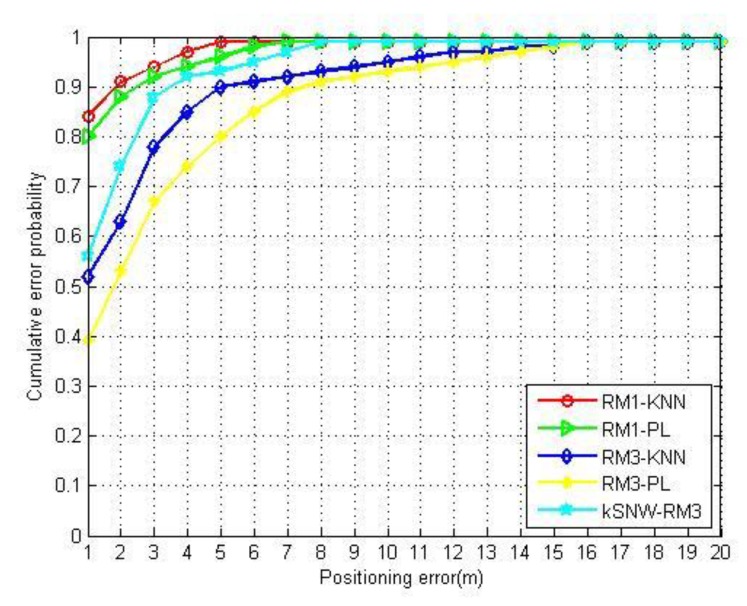
The positioning errors by different algorithms with different radio maps.

In order to compare the performance of different positioning algorithms, we used kSNW and KNN algorithms (*k* = 4), and the probabilistic positioning (PL) algorithm [25,26] 2526 under RM1 and RM3 to perform positioning tests. The results are shown in Figure 8. The PL algorithm calculates the conditional probability of RSSI samples and selects the RP with the maximum conditional probability as the estimated location. When using RM1 for positioning, both the KNN algorithm and the PL algorithm have achieved good positioning accuracy. In particular, the KNN algorithm can make full use of the sample statistical information to obtain the best positioning accuracy. When using RM3 for positioning experiments, the kSNW algorithm is better than the KNN algorithm and the PL algorithm. The probability of errors within 3 m by the proposed radio map is 88.30%, which is 10.30% and 21.20% higher than the KNN algorithm and PL algorithm, respectively. In the case of a random collection of unlabeled data, the RP’s distribution of the radio map is non-uniform, so the positioning accuracy of deterministic matching positioning algorithms such as KNN will inevitably decline. Notably, the positioning accuracy of kSNW-RM3 was reduced by up to 21% (at 1 m) compared to KNN-RM1, but the labeled samples were reduced by 75%.

In order to show the performance of the kSWN method, we also used the merging method proposed in this paper for localization. With the same positioning results, the kSWN algorithm takes about 7/8 less time than the merging method. The computation time of five experiments is shown in Table 4.

## 5. Conclusions

In the present study, a novel cost-effective method is proposed, merging the SLLE algorithm and GLP algorithm for building a radio map. This method noticeably lowers the calibration effort of location fingerprints and enhances the localization accuracy and robustness. This method first employs the SLLE algorithm for reducing the dimensions of RSSI samples and subsequently adopts a limited number of labeled location fingerprints for propagating the labeling data to those that are unlabeled. Lastly, the kSNW algorithm is developed for incorporating the local linear property to online positioning. In the experiment, we demonstrate that the proposed method has the acceptable positioning accuracy with the radio map construction under only 25% labeled fingerprints, and this has better results than the compared method. The kSNW algorithm has better adaptability than the KNN and PL algorithms with incomplete labeled fingerprints. This method reduces the time and labor cost of building a radio map by 75% while maintaining acceptable positioning accuracy. Future studies will focus on the optimization of the proposed method, for instance using the novel unsupervised learning method [27] and multi-tools fusion [28].

## Figures and Tables

**Table 1:** The received signal strength indication (RSSI) samples in an indoor environment.

Location	RSSI (dBm)												
	$AP_{1}$	$AP_{2}$	$AP_{3}$	$AP_{4}$	$AP_{5}$	$AP_{6}$	$AP_{7}$	$AP_{8}$	$AP_{9}$	$AP_{10}$	$AP_{11}$	$AP_{12}$	$AP_{13}$
$l_{m}\;$	−86	−73	−69	−63	−78	−58	−88	−65	−76	−56	−72	−80	−88
$l_{e}\;$	−86	−74	−70	−63	−76	−57	−87	−69	−79	−56	−74	−80	−89
$l_{s}\;$	−85	−73	−68	−61	−75	−55	−90	−70	−80	−57	−77	−82	−90
$l_{w}\;$	−85	−73	−68	−62	−77	−56	−89	−66	−76	−56	−78	−82	−90
$l_{n}\;$	−87	−75	−71	−64	−79	−60	−86	−64	−73	−55	−69	−79	−87

**Table 2:** KNN algorithm complexity comparison.

Localization Area	Status	RSSI Dimension	KNN Algorithm Complexity
Fourth floor of the YiFu building	Before dimensionality reduction	13	O(13N)
	After dimensionality reduction	4	O(4N)
Library	Before dimensionality reduction	19	O(19N)
	After dimensionality reduction	3	O(3N)

**Table 3:** The radio map construction scheme in the Library area.

Number	East–West Interval	North–South Interval	Labeled Fingerprint	Unlabeled RSSI Sample
DS 1	2 m	2 m	600	0
DS 2	4 m	2 m	300	300
DS 3	4 m	4 m	150	450
DS 4	6 m	4 m	100	500
DS 5	6 m	8 m	50	550

**Table 4:** The computation time of five experiments.

Number	Computation Time of kSWN	Computation Time of Merging Method
1	108 ms	951 ms
2	98 ms	871 ms
3	121 ms	784 m
4	78 ms	610 m
5	89 ms	709 ms
